# A Model of Core Emotional Needs and Toxic Experiences: Their Links with Schema Domains, Well-Being, and Ill-Being

**DOI:** 10.3390/bs14060443

**Published:** 2024-05-24

**Authors:** John Philip Louis, George Lockwood, Karen McDonald Louis

**Affiliations:** 1 Department of Psychology, UCSI University, Kuala Lumpur 56000, Malaysia; 2 Schema Therapy Institute Midwest, Kalamazoo, MI 49009, USA; glockwood@chartermi.net; 3 Louis Family Services, Inc., Fairview, TX 75069, USA; kmclouis@gmail.com

**Keywords:** core emotional needs, second order, schema domains, toxic experiences, well-being, ill-being

## Abstract

This study examined the second-order schema domains of Early Maladaptive and Adaptive Schemas based on recent trends and compared them with the five theoretical second-order schema domains commonly used in schema therapy. Using six international Eastern and Western community samples—Singapore (*n* = 628), Malaysia (*n* = 229), USA (*n* = 396), South Africa (*n* = 390), Nigeria (*n* = 364), India (*n* = 306)—confirmatory factor analysis showed that the four second-order domains of EMSs and EASs, which ran almost parallel with each other, were the most robust models calling into question the validity of the five domain model. Given the hypothesized links between schemas and needs, these four categories of EMSs and EASs represent four categories of toxic experiences and core emotional needs, respectively. These categories were supported empirically and are useful to parents as well as to clinicians as they approach child rearing and the treatment of clients in schema therapy from the vantage point of needs. These four categories of psychological core emotional needs, as well as toxic experiences, were found, as expected, to be linked with various measures of well-being and ill-being.

## 1. Introduction

Recent findings have shown that, among those under the age of five, an estimated 250 million children in low-income and middle-income countries (43%) are in danger of not achieving their developmental potential [1,2,3]. One primary reason suggested for this was failure to apply findings from science on how to increase the quality of nurturing care in the context of a home, the most pertinent source of such care [2]. This nurturing environment provides the psychological nutrients required for healthy development, and from the vantage point of schema therapy, these nutrients are known as core emotional needs [4,5,6]. Conversely, an environment that deprives children of these nutrients is associated with “toxic experiences” [4]. Repeated occurrences of toxic experiences in the home environment can result in the impairment of learning, behavior, and both psychological and physical health in childhood that will continue into adulthood [7].

Schema therapy is a fast-growing, effective approach to psychotherapy [8,9,10,11,12]. The core tenet of schema therapy is that a toxic and highly stressful environment, primarily in early childhood periods, will result in the development of strong, rigid negative schemas that will impede healthy development. Conversely, a nurturing environment, especially early on in life, will facilitate the development of strong positive schemas associated with healthy developmental outcomes. Negative schemas, formally known as Early Maladaptive Schemas (EMSs), and their counterparts, known as positive schemas, or more formally Early Adaptive Schemas (EASs), are made up of specific patterns of thoughts, emotions, beliefs, bodily sensations, and neurobiological reactions [4,6,13]. These schemas, a kind of mental structure, can be likened to giving a person a lens through which they view themselves and others, ultimately determining how they see and experience the world. If these mental structures are driven by strong, rigid EMSs, then views and events will be interpreted negatively, leading to an unhealthy behavioral disposition that will impair healthy development. On the contrary, if they are processed through the lens of strong EASs, then healthy views of themselves and others, as well as accurate interpretations of events, will be formed, which, in turn, will facilitate healthy development.

While schema therapy was originally developed for the treatment of borderline personality disorders [4], research has shown that EMSs, of which 18 have been identified to-date, are associated with a wide array of negative outcomes such as depressive symptoms [14], obsessive–compulsive disorder [15], bipolar disorder [16], eating disorders [17], unhealthy attachment styles [18], psychotic disorders [19], perceived injustice, anxiety [20,21], substance abuse [22], and post-traumatic stress disorder [23]. EASs, of which 14 have been identified to date, are a recent discovery [13], and the empirical support for these constructs is in the process of being replicated in other countries. Their links with well-being have only recently begun to be investigated [13,24,25,26].

One of the main goals of schema therapy is to weaken the strength of EMSs and simultaneously strengthen EASs. The latter makes up, according to the schema therapy mode, what is termed the “healthy adult” side of a client. The development of the healthy adult involves a therapist meeting a client’s core emotional needs by providing the missing psychological nutrients through a process called “Limited Reparenting” [4,10]. During this process, the therapist assumes the role of a nurturing parent, within the professional boundaries of the therapist–client relationship, and creates emotional experiences that meets needs that were thwarted in childhood. As a client’s core emotional needs are met, the EMSs gradually become weaker and the EASs stronger. Since the concept of core emotional needs is central to schema therapy, it is important to identify what these psychological nutrients are in order to increase understanding of the underlying mechanisms that make schema therapy effective. Table 1 shows the specific unmet needs linked with toxic experiences as well as the psychological nutrients or core emotional needs associated with each EMS and EAS, respectively. The process of meeting these needs is seen as the underlying mechanism contributing to healthy developmental outcomes in children as well as therapeutic change in patients in schema therapy.

The theoretical identification of these core emotional needs began when [4] initially identified the 18 negative schemas through years of clinical work with patients by observing reoccurring patterns of dysfunction. These patterns were explored in the context of the early toxic experiences with parents they were linked to as a way of determining the missing psychological nutrients. Young hypothesized that each EMS was a result of an unmet need and grouped the 18 negative schemas into 5 broader categories of “schema domains”. Each broader category defined the five major types of unmet needs that were seen as counterparts to what have been termed core emotional needs [4,5]. This was performed to both serve as a clinical heuristic and to further elucidate the nature of these needs [4,6]. These categories of unmet needs linked with five major types of toxic experiences were labeled Disconnection and Rejection, Impaired Autonomy and Performance, Impaired Limits, Other-Directedness, and Overvigilance and Inhibition [4]. However, Bach et al. [27] identified four categories that were slightly different, and these were labeled as Disconnection and Rejection, Impaired Autonomy and Performance, Impaired Limits, and Excessive Responsibility and Standards.

While unmet needs are conceptualized on both the primary level (the 18 first-order EMSs being seen as an expression of 18 distinct needs) and the secondary level (the 5 broader categories of schema domains), in this paper, we will be focusing on the latter. Young’s 18 EMSs have been repeatedly validated empirically; however, there has been little empirical support for the five groupings of schema domains as proposed by Young et al. [4]. Numerous studies have been conducted, and the literature has been written assuming that the five groupings of the 18 EMSs are supported empirically when this is not the case, for example, Mavroeides et al. [28], Arntz et al. [29], Shorey et al. [30], Corral and Calvete [31], Flink [32], Saggino et al. [33]. This is, therefore, an important issue to address. In recent years, only Siahmoshtei [34] provided weak preliminary support for the five groupings or domains, but these analyses were performed qualitatively using a very small sample size of eight subjects and quantitatively using only thirty-three subjects. On the contrary, over the last two decades, at least ten studies have found support for a four domain model—Macik & Macik [35], Thimm [36]; Aloi et al. [37], Yalcin [38], Bach et al. [27], Sakulsriprasert et al. [39], Samuel and Ball [40], Calvete [41], Hoffart et al. [42], Cecero et al. [43], Lee et al. [44]. A three domain model was also found by Thimm [36], Cui et al. [45], and Schmidt et al. [46], as well as a one-factor model for all 18 EMSs by Kriston et al. [47]. Of note, none of the above studies found robust support for the five domain model as proposed by Young et al. [4]. Given the continued reliance on the five-domain model, it will be helpful to test this directly in relation to these alternative models.

Given that the five second-order schema domains of EMSs were postulated to represent categories of patterns that result from psychological toxins that lead to unmet needs, it was also proposed in schema therapy that the second-order domains of EASs (counterparts to the second-order domains of EMSs) represent categories of patterns that are an outgrowth of needs that are met through the provision of psychological nutrients [4,5,6]. This paper is based on that hypothesis. This hierarchal model of EASs has been tested by Louis et al. [5], and a four-second-order structure that ran almost parallel to the four-second-order structure of EMSs was identified. These categories were labeled as Connection and Acceptance, Healthy Autonomy and Performance, Reasonable Limits, and Healthy Standards and Reciprocity [5] (see right side of Table 1). Moreover, it was found that EASs were not merely positive versions of EMSs falling on the same continua. Louis et al. [48] showed statistically that EMSs and EASs were independent but related constructs. Given that they are independent constructs, the emergence of a complimentary set of second-order structures provides two distinct lenses through which to view core emotional needs. If such categories can be robustly supported, knowledge would be advanced in three important areas. First, this would potentially add to our understanding of the underlying mechanisms that bring about the deeper-level changes that are associated with the effectiveness of schema therapy. Second, this clearer understanding will help therapists to more precisely identify and effectively meet the core emotional needs that may lie at the root of their clients’ ongoing struggles. Third, this will help parents and caregivers better understand the full range of core emotional needs arising through the course of development. This will also help parents with how to be aware of and minimize psychological toxins and better provide the key psychological nutrients that will help their children thrive. A clearer delineation of the categories of core emotional needs and psychological toxins would provide a more comprehensive and empirically valid blueprint for both caregivers and clinicians of the kind of nurturing environment needed for, and the type of toxic environment not conducive to, positive developmental outcomes in children and clients.

## 2. The Present Research

The first aim of this study was to test several models of second-order schema domains of EMSs associated with toxic experiences using multi-cultural samples, both Eastern and Western, as well as samples from the developing and developed world. The models tested include the Young et al. [4] five-schema domain model, a four-schema domain model by Bach et al. [27], and a three-schema domain model obtained by Schmidt et al. [46] and Cui et al. [45]. If a four domain model proves to be the most robust, which has been the trend to-date, then an argument for the revision of the five-domain model as proposed by Young et al. [4] is needed as this model is still assumed to be valid by many clinicians and researchers. Given the trend in the research, it was hypothesized that a four-category model of unmet needs by Bach et al. [27] associated with toxic experiences would emerge as the most robust.

The second aim of this study was to test the second-order schema domains of the EASs using the same diverse samples as that used for EMSs outlined above. The empirical identification of EASs is a relatively recent discovery [13]; therefore, there is only one second-order model of EASs, which consists of four factors [5]. It was hypothesized that this four-factor model would meet acceptable fit indices.

The third and final aim of the study was to see if the second-order domains of EMSs and EASs are linked to various measures of well-being and ill-being. It was hypothesized that the core emotional needs (represented by the second-order factors of EASs) would be linked to well-being and that toxic experiences (represented by second-order factors of EMSs) would be linked to ill-being.

## 3. Method

### 3.1. Samples

All participants were volunteers of an NGO headquartered in the USA. Each of the NGOs were contacted by the principal investigator, who was a former board member of one of the NGOs, to see if their volunteers would be interested in taking part in this study with incentives to participate in online parenting workshops without charge conducted by the principal investigator. In Singapore, participants were given a free copy of the first author’s book on parenting as an incentive for completing the questionnaires since this workshop had already been conducted in the past. Data for this study were drawn from volunteers for these NGOs from six countries during two different periods. The first was drawn in 2016 from two Southeast Asian countries, namely Singapore and Malaysia. The second was based on online data collection during the COVID-19 outbreak in 2019 from the USA, South Africa, Nigeria, and India. The demographic characteristics of both sets of samples are shown in Table 2 and Table 3. In total, there were six non-clinical English-speaking community samples. The sample size (*n*), mean age, and standard deviation (SD) were as follows: Singapore (*n* = 628), 46.22 years (SD = 22.34); Malaysia (*n* = 229), 41.40 years (SD = 17.40); USA (*n* = 396), 43.69 years, (SD = 9.12); South Africa (*n* = 390), 42.11 years (SD = 6.79); Nigeria (*n* = 364), 45.7 years, (SD = 7.19); India (*n* = 306), 42.39 years (SD = 7.67). For the Singapore and Kuala Lumpur samples, participants came together physically in one setting where the questionnaires were administered. After completion, data of their responses were given to the first author and his assistant, who then scanned the data into digital format. For the other four samples, data were collected online after participants completed giving their responses. All participants were 18 years and above, and no one was excluded based on race, color, gender, or religion. All of them also had an adequate command of the English language, and this requirement was made clear to all volunteers by their respective polling of heads in each city. The objectives of this research, the voluntary nature of their involvement, the signing of a consent form, the estimated amount of time needed for the completion of the questionnaires, as well as the confidentiality of information were made clear by the first author through emails and announcements to these affiliates who then disseminated them to the volunteers. Ethical considerations were in line with standards advocated by the American Psychological Association and the British Psychological Society. Approval to conduct this research study was given by the ethics committee in each NGO. Volunteers were drawn from populations in communities comprising professionals, singles, students, the married, and parents.

Apart from the USA, all these countries were part of the former British colonies in which the English language was and still is one of the main media of instruction. As a result, it was not difficult to find English-speaking volunteers. Of note, outside of the USA and the UK, India and Nigeria are some of the largest English-speaking countries in the world. This introduces a huge advantage of not having to translate all the psychological instruments, as this would otherwise involve rigorous amounts of work to validate these instruments in these populations.

For the online survey, a Google Survey Form was created. Online participants were requested to take the survey together at a particular date and time, which was about three weeks before the online parenting workshops. Completing the questionnaires online together would enable them to raise any questions via “chat box” or by asking the first author and his assistants verbally as they were also present. The online survey was formatted in such a way that all questions had to be answered by each participant. However, if they were not able to do so, they would be allowed to complete the survey at their own convenience. For all questions to be answered, any unanswered questions would trigger an alert to remind the participant to go back and provide a response. As a result, there were no missing values from any unanswered items. Participants who were distressed in any way by the questions were allowed to opt out of taking the entire survey, and they would not be penalized with exclusion from the online workshops offered as incentives. The entire survey, on average, took about 45 min to 1 h to complete. Data were then sent to the first author and his assistant. In the data, all fields that were able to identify a participant were removed for confidentiality purposes.

### 3.2. Instruments

The following instruments were administered to the four online samples, namely the USA, South Africa, Nigeria, and India, as well as in-person samples in Singapore and Malaysia (Kuala Lumpur).

### 3.3. Negative Schemas or EMSs

The latest version of the instrument that measures 18 EMSs or negative schemas is known as the YSQ-S3 [49]. There are 90 item items in total and responses are based on a six-point Likert scale, which ranged from a score of 1 (Completely untrue of me) to a score of 6 (Describes me perfectly). Item examples are “I find myself clinging to people I’m close to because I’m afraid they’ll leave me” (Abandonment negative schema), and “I don’t have people to give me warmth, holding, and affection” (Emotional Deprivation negative schema). EMSs have also been identified in the following countries—Norway, the United States, China, Australia, Korea, Turkey, and the United Kingdom [42,43,45,50,51,52]. More recently, the psychometric properties of EMSs have been shown in a study conducted in Korea [53], where all 18 schemas were shown to correlate positively with anxiety and depression, which were measured using the subscales of the Symptom Checklist [54]. In Germany [55], the EMSs were validated using a community as well as a smaller clinical sample, and the internal consistency of 17 subscales was >0.70 (except the Entitlement schema, which was 0.67). In Denmark [56], all 18 EMSs were supported and had an internal consistency of alpha > 0.7.

### 3.4. Positive Schemas or EASs

The theoretical framework of positive schemas was set out by Lockwood & Perris, 2012, and later, 14 EASs were empirically identified by Louis et al. [13] in multicultural community samples and developed into a scale known as the Young Positive Schema Inventory (YPSQ). The instrument consists of 56 items, and each item requires a response based on a six-point Likert scale with a score from 1 (Completely untrue of me) to a score of 6 (Describes me perfectly). Item examples are “I am usually OK with not getting my way in a group decision” (Empathic Consideration positive schema) and “If I make a mistake, I can usually forgive myself; I don’t feel that I deserve to be punished” (Self Compassion positive schema). The YPSQ was validated and demonstrated significant correlations with measures of distress such as depression, anxiety, and stress [13]. EASs from the YPSQ have also been shown to be separate but related constructs with EMSs as opposed to being the same measure but on the opposite side of a continuum [48]. The reliability values for 12 EASs were >0.76, and for the other two, they were >0.62 [13].

The following instruments were administered online to these four samples only—the USA, South Africa, Nigeria, and India.

### 3.5. Dark Triad

Jones and Paulhus [57] validated the Dark Triad (DT) instrument. It consists of 27 items, each rated on a five-point Likert-type scale that ranges from 1 (Strongly disagree) to 5 (Strongly agree). Acceptable reliability values were found for each of the three subscales—Machiavellianism, α = 0.71; psychopathy, α = 0.77; narcissism, α = 0.80. Item examples are as follows:

“I tend to want others to admire me” (narcissism), “I tend to not be too concerned with morality or the morality of my actions” (psychopathy), and “I have used deceit or lied to get my way” (Machiavellianism). Concurrent validity was demonstrated with the Christie–Geis Machiavellianism scale [58] (correlation value of 0.68); psychopathy of the DT with Self Report Psychopathy Scale [59] (correlation value of 0.78); narcissism of the DT with Narcissistic Personality Inventory [60] (correlation value of 0.70).

The following instruments were administered in-person to the Singapore and Malaysia (Kuala Lumpur) samples only.

### 3.6. The Mini-International Personality Item Pool (Mini-IPIP)

The Mini-IPIP consists of 20 items that measure the Big Five personality traits, namely Agreeableness (“Sympathize with others’ feelings”), Conscientiousness (“Get chores done right away”), Extraversion (“Am the life of the party”), Intellectual Openness (“Have a vivid imagination”), and Neuroticism (“Have frequent mood swings”). Responses are based on a five-point Likert scale, which ranges from a score of 1 (Very inaccurate) to a score of 5 (Very accurate). The Mini-IPIP has been demonstrated to have high test–retest correlations in the short term (0.62 to 0.87) and long term (0.68 to 0.86) [61,62].

### 3.7. The Gratitude Questionnaire–6 (GQ-6)

The GQ-6 consists of six items, and each measures the disposition to experience gratitude. Responses are based on a seven-point Likert scale, from 1 (Strongly disagree) to a score of 7 (Strongly agree). An item example is “I have so much in life to be thankful for”. The psychometric values of the GQ-6 scale have been demonstrated robustly, for example, with impaired sleep quality (*r* = −0.11 to −0.29), positively with pre-sleep cognitions (*r* = 0.21) [63], and other measures of well-being like positive and negative schemas [13].

### 3.8. Depression, Anxiety, and Stress Subscales (DASS-21)

The DASS-21 consists of 21 items measuring three types of emotional distress: depression, “I couldn’t seem to experience any positive feeling at all”; anxiety, “I was worried about situations in which I might panic and make a fool of myself”; and stress, “I tended to over-react to situations”. Responses are based on a four-point Likert scale, from 0 (Did not apply to me at all) to 4 (Applied to me very much or most of the time). High concurrent validity (*r* > 0.50) was shown by Antony et al. [64] with the Beck Depression Inventory and the State-Trait Anxiety Inventory-Trait version [65].

### 3.9. Satisfaction with Life Scale (SWLS)

The SWLS [66] consists of five items which measure the degree of satisfaction in one’s life and is based on a seven-point Likert scale from 1 (Strongly disagree) to 7 (Strongly agree). An item example: “The conditions of my life are excellent”. A strong test–retest reliability coefficient of 0.82 was reported by Diener, Emmons, Larsen, and Griffin [67], as well as a strong negative correlation with the Beck Depression Inventory [68].

## 4. Procedures and Statistical Analyses

For all analyses, IBM SPSS Statistics 23 [69] and MPlus 8 software were used [70]. There were no missing data for the online participants from the four countries—the USA, South Africa, Nigeria, and India—since the responses were formatted in such a way that participants would either respond to all the items or opt-out entirely. For the other two countries where the administration of the questionnaires was performed in-person, participants were merely encouraged to answer all the questions. However, the missing data were very small—0.06% for Singapore and 0.07% for Malaysia. Schafer [71] asserted that the impact of missing data of 5% or less is inconsequential, which was the case for these two samples.

The first aims of this study were the investigation of the five schema domains, or the second order of EMSs, proposed by Young et al. [4]; the hypothesized four schema domains as found by Bach et al. [27]; and the three-schema domains proposed by Schmidt et al. [46] and Cui et al. [45]. It was determined a priori to only accept models with all 18 EMSs or all EMSs identified at the time of the study since all 18 EMSs have been identified in numerous studies all over the world [4,6,13]. It was also determined a priori to accept the four second-order EMSs model by Bach et al. [27] as being the most robust as this work used large clinical and non-clinical samples. As far as the three second-order EMS models, both models from Cui et al. [45] and Schmidt et al. [46] were also investigated.

Thereafter, these models would be subjected to confirmatory factor analysis (CFA) using all six samples to test their robustness. Following CFA, the most robust model would then be subjected to multi-group CFA (MGCFA). All six international sample sizes were >200—Singapore (*n* = 628), Malaysia (*n* = 229), the USA (*n* = 396), South Africa (*n* = 390), Nigeria (*n* = 364), and India (*n* = 306), and as a result, according to Tabachnick and Fidell [72] as well as Floyd and Widaman [73], the CFA in each sample will be robust against violations of skewness and kurtosis. CFA and MGCFA were conducted using a weighted least-squares means and variance adjusted estimation (WLSMV) algorithm in order to take into account the categorical nature of the response scales [74]. Three broad fit indices categories were used—first, the Absolute fit indices, which are measures of how well the model fits when there is no model at all, were used [75]. An example of such a fit was the Root Mean Square Error of Approximation, where a good fit is obtained when RMSEA <0.05, a reasonable fit is obtained when 0.06 < RMSEA < 0.08, a mediocre fit is obtained when 0.08 < RMSEA < 0.10 [76]. The second measure of fit indices used was the Incremental fit indices, and examples of such indices used in this study would be the Comparative Fit Index (CFI) and the Tucker–Lewis Index (TFI). For both CFI and TFI values, ≥0.95 is regarded as a good fit, and ≥0.90 is an adequate fit. The final fit indices used were the Parsimonious fit which was obtained by dividing the chi-square values (*X*^2^) with degrees of freedom (*df*). Values when *X*^2^*/df* < 2 to 3 were regarded as a good fit. However, scales with high numbers of items and factors generally lead to a poorer fit [73]. This was evident from three studies, namely Bach et al. [27], Baranoff et al. [50], and Kriston et al. [55], where 18 EMSs with 90 items yielded CFI values that were slightly below the 0.90 threshold with values of 0.84, 0.87, and 0.85, respectively. Thus, for acceptable fit, slightly more relaxed values for indices may be considered, and since a large number of items was used in the EMSs and EASs scales, it was determined a priori to accept the lower bound of fit values as fitting in this context.

The second aim of this study: Since the identification and empirical support for EASs has only been performed recently [13], only one study to date has investigated the second orders of EASs where three, four, five, and six factors were tested on Eastern and Western samples [5]. Results to date showed that this four-factor model of EASs, consisting of all 14 EASs, was the most robust one, with no other alternative model tested or proposed. Notwithstanding this, this four-factor model by Louis et al. [5] would be tested using CFA on the six international samples to see if it will have acceptable fit indices and statistics.

The third aim of this study: It was hypothesized that the core emotional needs would be represented by the second-order factors of EASs and that toxic experiences would be represented by second-order factors of EMSs. Here, investigations were conducted on the associations between second-order factors of EMSs and EASs and measures of well-being and ill-being using combined Singapore and Malaysia samples (*n* = 628, *n* = 229). Combined samples were used in order to increase the sample size as much as possible to enable a more precise determination of the regression parameters. These investigations were performed using Pearson’s correlations with *p*-values tested at 0.001, 0.01, and 0.05 levels. Threshold guidelines were adopted from Cohen [77] for effect sizes considered small when *r* = 0.10, medium when *r* = 0.30, and large when *r* = 0.50. Using combined samples of four countries (the USA, South Africa, Nigeria, and India, *n* = 1456), Pearson’s correlations were also conducted with the DT scale. However, before Pearson’s correlation analyses were conducted, certain assumptions [78] needed to be satisfied. These were as follows: (1) the variables need to use a continuous scale, (2) the two variables should have a linear relationship which can be ascertained using a scatterplot, (3) there should be no spurious outliers, (4) the variables should be normally or near-to-normally distributed.

Following this, linear regressions were also conducted. Again, certain assumptions [78] must be satisfied before results can be interpreted as reliable, and these are as follows: (1) the dependent variables must be on a continuous scale; (2) there must be two or more independent variables; (3) these variables should have a linear relationship, which can be checked using a scatter plot; (4) data should have homocedasticity, which means that the line of best fit should not be dissimilar as data points move across the line either in a positive or negative direction; (5) data should not contain variables that are highly correlated; (6) there should be no spurious outliers; (7) there should be normal distribution of the residual (errors).

Upon satisfying all these assumptions, linear regression analyses with each of the three DT subscales using combined four samples (*n* = 1456) were performed. Using combined Singapore and Malaysia samples, a separate set of linear regression analyses was also conducted using the same measures of well-being and ill-being as dependent variables with the best second-order models for EMSs as independent variables. This was repeated separately with the second-order model of EASs. This enabled investigations on the impact of the second order of EMSs and EASs on these scales. Two multiple regressions were estimated for each outcome: (1) all categories of the most robust second-order domains of eMSs were entered as predictors in each multiple regression; (2) all categories of the second-order domains of EASs; (3) these were repeated with second order domains of EASs and EMSs entered as single predictors representing core emotional needs and toxic experiences, respectively.

## 5. Results

Our first aim: the three second-order EMS models that were subject to investigation were the five-factor one proposed by Young et al. [4], the four-factor from Bach et al. [27], and a three-factor model from Cui et al. [45], and Schmidt et al. [46]. However, both of these three second-order models did not contain all the EMSs that had been identified at their respective times of testing. Specifically, the model that emerged from Cui et al. [45] did not contain the EMS of Subjugation. Schmidt et al.’s [46] model also did not include the EMSs of Entitlement and Insufficient Self-Control. Since it was determined a priori to accept a model with all EMSs identified at their time of testing, both these three-schema domain models were rejected. The other two models with four- and five-schema domain models were tested using CFA, and Table 4 shows their results. All fit indices were close to each other and were acceptable, although Young’s five domains had fit CFI and TLI indices that were slightly less robust in samples from the USA and Singapore compared to the four-factor model. The four-factor model from Bach et al. [27] was, therefore, concluded as having the most robust model. This four-factor model was then subjected to MGCFA, the most stringent test for invariance. Results in Table 5 showed that invariance was obtained for this four-factor model on all seven levels. The four second-order domains of EMSs obtained by Bach et al. [27] were labeled Disconnection and Rejection, Impaired Autonomy and Performance, Impaired Limits, and Excessive Responsibility and Standards. Since each EMS was theorized to be a specific category of unmet need, these four categories of EMSs represented broader categories of unmet needs or categories associated with toxic experiences [4,27].

Our second aim: the four-factor model of EASs was tested using CFA on all six international samples. Results in Table 6 showed acceptable fit indices on all six international samples. Table 7 showed invariance on all six out of the seven levels, showing sufficient cross-cultural support for this model. These four second-order EAS domains were labeled as Connection and Acceptance, Healthy Autonomy and Performance, Reasonable Limits, and Healthy Standards and Reciprocity [5,79], and since each EAS is a result of a specific need met, these four categories represented four broader categories of core emotional needs [4,6,13].

Our third aim—The assumptions for Pearson’s correlations were satisfied based on the following observations and analyses: (1) All the instruments used in this study had Likert scales, and those with five or more categories can be used as continuous without any harm to the analysis [80,81]. In this study, all instruments had at least five categories, except for DASS-21, measuring depression, stress, and anxiety, which had four categories. However, even with four categories or less, the sum or mean of the ordinal variables across a set of questions was taken, and this resulted in a number of categories that were much higher than the ordinal Likert scales they were calculated from, which resulted in an approximate continuous variable. Therefore, all variables were used as continuous variables. (2) The variables had linear relationships, which was ascertained from observation of scatterplots where straight lines between variables sloped either in the positive or negative direction but not in a non-linear pattern. (3) The interquartile range (IQR) was used to determine outliers, according to Tukey [82]. A datapoint is considered an extreme outlier if it is within the 25th and 75th percentiles of data distribution [82]. Data from the various samples showed that there was only one variable with two outliers, but these had a negligible effect on the overall results given the large sample size and, therefore, it was concluded that there were no spurious outliers. (4) Results, using all six samples combined (*n* = 2313), revealed values of skewness that ranged from −0.64 to 0.58, and that of kustosis ranged from −0.25 to 0.26. Hair et al. [83] argued that data can be regarded as normally distributed if the value of skewness is between −2 and +2 and the value of kurtosis is between −7 and +7. The values obtained from our samples were well within these ranges, and therefore, the distribution of data was considered normal. Given that the above assumptions were satisfied, Pearson’s correlations were conducted between categories of toxic experiences (the four second-order domains of EMSs) and the four categories of core emotional needs (four second-order domains of EASs), where all six samples were combined (*n* = 2313). Results showed a negative linear relationship between each of the four core emotional needs and toxic experiences, as shown in Table 8. For the most part, the effect sizes were medium to large. Also, all correlations were <0.85, indicating no significant overlap between them, supporting the independence of constructs.

With measures of well-being and ill-being, the categories of core emotional needs showed significant negative correlations with IPIP Neuroticism, depression, anxiety, and stress but positive correlations with IPIP Conscientiousness, IPIP Extraversion, IPIP Intellect, Gratitude, and SWLS (see Table 9). As expected, also from Table 9, the four categories of toxic experiences showed significant relationships in the opposite direction with these measures.

Table 10 shows the negative correlations between Machiavellianism, narcissism, and psychopathy and the four categories of core emotional needs. All four categories had a negative relationship with Machiavellianism and psychopathy but an unexpected positive relationship with narcissism. All four categories of toxic experiences had a positive relationship with Machiavellianism and psychopathy, but narcissism did not correlate as expected with Impaired Autonomy and Performance, as well as with Disconnection and Rejection, but correlated positively with the toxic experiences of Impaired Limits, and Excessive Responsibility and Standards.

The various assumptions upon which linear regression analyses were based were then tested, and the following observations and results were obtained: (1) the dependent variables were on a continuous scale (as was the case for the assumption for Pearson’s correlations); (2) there were more than two independent variables; (3) the relationships between the variables were linear from observation of the scatterplots (as was the case for the assumption for Pearson’s correlations); (4) data had homocedasticity where the lines of best fit were not disimiliar as data points moved in either positive or negative direction, and this was determined using standardized residual plots against the unstandardized predicted values, and residual normality was checked using Q-Q plots; (5) data were not highly correlated with each other seen from the values of correlations with each other, which were all <0.80 [83]; (6) as mentioned under the assumptions of Pearson’s correlations, there were no spurious outliners; (7) the distribution of the residual (errors) were normal. Given that all assumptions were satisfied, linear regression analyses were conducted with four categories of core emotional needs and toxic experiences, all entered as separate predictors, with measures of well-being and ill-being as dependent variables.

Results in Table 11 showed that the core emotional need for Connection and Acceptance had a positive and significant outcome on IPIP Agreeableness, IPIP Extraversion, IPIP Intellect, Gratitude, and SWLS. Healthy Autonomy and Performance had negative correlations with IPIP Extraversion and IPIP Neuroticism but significant positive correlations with Gratitude and a negative one with Anxiety. Other significant relationships were the negative ones between Reasonable Limits and Depression but a positive one with IPIP Conscientiousness. Healthy Standards and Reciprocity had a negative relationship with Stress and IPIP Neuroticism. IPIP Neuroticism had negative relationships with two of the core emotional needs, but the other IPIP traits had both positive and negative relationships with the four categories of core emotional needs. However, when all these four categories were entered as one predictor (Table 12), they correlated positively and significantly with all IPIP personality traits except for IPIP Neuroticism. This showed that each individual predictor of core emotional needs was not robust in their relationship with IPIP traits as when they were combined together as a single predictor. When combined, the core emotional needs showed expected negative correlations with depression, anxiety, and stress as well as expected positive ones with Gratitude and SWLS.

Relationships between toxic experiences and IPIP well-being and ill-being are shown in Table 13. Disconnection and Rejection, as expected, had negative relationships with IPIP Agreeableness, IPIP Extraversion, Gratitude, and SWLS but a positive one with IPIP Neuroticism, Depression, Anxiety, and Stress. Impaired Autonomy and Performance had a positive relationship with Depression, Anxiety, and Stress, as well as with IPIP Neuroticism. Impaired Limits, as expected, had a negative relationship with IPIP Conscientiousness but positive with IPIP Extraversion. Excessive Responsibility and Standards had unexpected positive correlations with Gratitude and SWLS. However, the expected directions were obtained when all four categories of toxic experiences were combined, as shown in Table 14, where they had expected negative relationships with all four IPIP personality traits but a positive one with Neuroticism. All four categories of toxic experiences also showed positive relationships, as expected, with depression, anxiety, and stress, and also an expected negative one with Gratitude and SWLS.

With DT and each category of core emotional needs, results in Table 15 showed that Healthy Autonomy and Performance had the most significant negative relationships with all three subscales of the DT scale. Healthy Responsibility and Reciprocity also had a negative relationship with narcissism. Connection and Acceptance as well as Reasonable Limits have an unexpected positive relationship with Narcissism. However, when all four categories of core emotional needs were combined (see Table 16), they had a negative relationship with Machiavellianism and psychopathy, although the R^2^ values were low. However, the four categories of core emotional had an unexpectedly significant positive relationship with narcissism.

The linear regression results between the four categories of toxic experiences and DT scales are shown in Table 17. Disconnection and Rejection showed a positive relationship with Machiavellianism and psychopathy; Impaired Limits showed positive relationships, as expected with all three of the DT subscales. Impaired Autonomy and Performance showed unexpectedly negative relationships with all three subscales of the DT scale. Excessive Responsibility and Standards showed a weaker positive relationship with narcissism. However, when combined, as shown in Table 18, the expected positive directions emerged between the DT subscales and all four categories of toxic experiences combined. The R^2^ values, on the whole, for many relationships, were >0.1, which, according to Hair et al. [84], is satisfactory. Specifically, the individual and collective categories of core emotional explained significant proportions of variances with values of R^2^ > 0.1 in measures of well-being, ill-being, and most of the IPIP traits but not in any of the DT subscales. However, the individual and collective categories of toxic experiences explained significant proportions of variances with values of R^2^ > 0.1 in the measures of well-being, ill-being, most of the IPIP traits, as well in two subscales of DT, namely Machiavellianism and psychopathy.

## 6. Discussion

This study set out to investigate the various models of schema domains for both the EMSs and EASs. Young et al.’s [4] five-domain model of EMSs was tested using CFA, and it was found to not be as robust as the four-domain model of Bach et al. [27] (Table 4). MGCFA of the Bach et al. [27] model showed this to be supported at all seven levels of invariance and to be a consistent and cross-culturally acceptable one in all of the six international samples. Moreover, given the trend from over ten other studies—Macik and Macik [35], Thimm [36], Aloi et al. [37], Yalcin [38], Bach et al. [27], Sakulsriprasert et al. [39], Samuel & Ball [40], Calvete [41], Hoffart et al. [42], Cecero et al. [43], Lee et al. [44]—greater support was found for the four domain model of EMSs than Young et al.’s five [4]. As a result, the robustness of the five-schema model of Young et al. [4] has to be questioned. The testing of the four-domain model of EASs, originally proposed by Louis et al. [5], was shown to also be a robust model with acceptable CFAs (see Table 6) and MGCFA (see Table 7) fit indices and fit statistics. MGCFA showed invariance in six out of the seven levels of invariance, sufficient to show multi-cultural support from the six international samples.

Each EMS, according to schema therapy [4], involves a distorted view of oneself, others, and the world and results from a specific unmet need earlier in life (Table 1). Thus, each of the 18 EMS corresponds to a specific unmet need, resulting in 18 specific unmet needs. The higher-order framework for these 18 EMSs summarizes and organizes these patterns into four categories of unmet needs or toxic experiences labeled Disconnection and Rejection, Impaired Autonomy and Performance, Impaired Limits, and Excessive Responsibility and Standards. Conversely, each first-order EAS was theorized to involve a specific healthy view of self, others, and the world, which resulted from a specific need met earlier in life. Therefore, the four second-order EASs reflected categories of constructs viewed as representing broader categories of needs that were met (i.e., core emotional needs). These categories of core emotional needs were labeled as Connection and Acceptance, Healthy Autonomy and Performance, Healthy Limits, and Healthy Standards and Reciprocity. The four categories of core emotional needs and toxic experiences were correlated with each other, and results showed that their effect sizes were way below 0.60, demonstrating support for their independence, as demonstrated earlier by Louis et al. [48].

While overall toxic experience and core emotional needs showed expected correlations with other measures of well-being and ill-being, certain relationships that had emerged from this study are worthy of note. For example, Healthy Autonomy and Performance had the strongest statistically significant negative relationships with each of the three subscales of the DT scale—Machiavellianism, narcissism, and psychopathy (see Table 15). Doerfler et al. [85] also showed that Mahchiavellianism, narcissism, and psychopathy of the DT are associated with a weak sense of self—difficulty figuring out one’s own personality, goals, feelings and putting more weight onto other people’s concerns more than one’s own. These findings suggest that these problems have important links to a need for healthy autonomy. Another important pattern was the link between Impaired Limits and all the DT subscales. This can be explained by the negative EMS of Entitlement that is a significant part of this Impaired Limit category which drives behaviors that disregard rules [4,48,86].

Connection and Acceptance and Disconnection and Rejection had unexpected results in the opposite direction than narcissism had. This may have risen due to poor construct validity reflected by items representing narcissism such as “I am an average person”, “I insist on getting the respect I deserve”, and “I feel embarrassed if someone compliments me”. It could be argued that these items do not represent pathological narcissism. Adaptive functioning seems likely to be reflected in moderately high scores on these items. Thus, it can be viewed as healthy to not think of oneself as average, to feel strongly about being treated with respect, and to feel comfortable with, and even enjoy, receiving a compliment. Pathological narcissism is reflected in extremes of grandiosity, distrust, self-centeredness, and antagonism. Items that zero in on exhibitionism, lack of humility/modesty, interpersonal dominance, negative affect, distrust, selfishness, and a need for attention and recognition [87] are likely to be better measures of this construct.

It was also interesting to see that toxic experiences explained more significant proportions of variances with values of R^2^ > 0.1 in the subscales of DT than the core emotional needs, implying that unmet needs of EMSs or toxic experiences have more predictive power in relation to the DT subscales of Machiavellianism and psychopathy than the core emotional needs. In other words, results from this study indicated that the effects of not meeting needs have a separate and greater predictive power than the effects of meeting the needs when it comes to relationships with the DT constructs of Machiavellianism and psychopathy. However, categories of core emotional needs and toxic experiences, individually and collectively, accounted for significant proportions of the variances with values of R^2^ > 0.1 in measures of well-being, ill-being, and most of the IPIP traits.

In summary, the four categories of core emotional needs were positively correlated with Gratitude, SWLS, and negatively with measures of emotional distress (depression, anxiety, and stress), and conversely, the four categories associated with toxic experiences were associated negatively with measures of well-being (Gratitude, SWLS) and positively with scales measuring emotional distress. It is important to note that both these experiences are related to each other in that an increase in one will cause a decrease in the other, but not to the same degree. Both constructs are separate though related to each other, as shown by Louis et al. [48] and this study. Meeting these core emotional needs does not automatically lead to a reduction in toxic experiences to the same degree. This suggests that therapeutic leverage will be increased by approaching the process of meeting core emotional needs and reducing toxic experiences as both overlapping/synergistic and, at the same time, distinct phenomena. Parents need to be intentional about both what kind of emotional climate to promote at home and what to avoid. For example, parents should not just avoid Disconnection and Rejection, but they should also be proactive in forging the kind of emotional connection with their children that will help meet the core emotional needs for Connection and Acceptance. This comes, for example, from spending nurturing times with their children, including the consistent one-on-one time that is helpful in, among other things, countering perceived favoritism among siblings. With respect to the interaction between negative and positive schemas, it will be helpful for parents and therapists to keep in mind that it will take a significant number of positive experiences to undo or counteract a single negative one and that this is especially true of schemas within the Disconnection/Rejection and Connection/Acceptance Domains. In other words, relatively infrequent moments of Degradation and Rejection can have a disproportionately damaging impact on a sense of Connection and Acceptance. It is important for parents to avoid eroding a sense of autonomy, agency, and competence by, for example, not being overcontrolling and imposing their agenda. At the same time, it is important for parents to help their children make better, age-appropriate decisions; empower them to take responsibility for their own lives; and believe in them. Avoiding these toxic experiences and meeting these core needs will both contribute to the minimization of Impaired Autonomy and Performance and the fostering of Healthy Autonomy and Performance. With respect to limit setting, it is best for parents to not be lax and neglectful, leaving their children to their own devices and impulses. On the positive side, it is important for parents to provide reasonable limits, be attuned to the child in their environment, and provide the wisdom and guidance that will help them internalize these in an atmosphere of love and patience. Finally, it will be helpful for parents to avoid imposing unrealistic expectations and an excessive sense of responsibility and, thereby, burden a child with pressure to excel or a pattern of self-sacrifice and guilt. In addition to this, again, on the adaptive side, children will need help in developing well-attuned expectations, a healthy balance between work and play, and a healthy balance between giving to others and taking care of one’s self—the core emotional need for Healthy Standards and Reciprocity [79].

These results will also have important implications for therapists who will be better positioned to link the various psychological problems that a client may face with a specific unmet need in childhood and not just focus on weakening the negative schemas linked to the client’s problem. A dialogue can then take place to uncover ways this need was deprived, and a deeper understanding of the nature of the client’s relationships with their past caregivers developed with respect to the good things that were missed as distinct from the toxic experiences that occurred. This will also deepen the therapist’s understanding of how to meet these needs in the “here and now” through the limited reparenting process. For example, a client may come to a therapy session feeling depressed as a result of not feeling “good enough” to be accepted at his workplace and/or by his family members. While the therapist can link these unhealthy messages with a particular set of EMSs (such as defectiveness and emotional deprivation), the therapist is now able to associate these two EMSs with the toxic experiences of Disconnection and Rejection. The therapist will also be able to know that the most suitable antidote to this toxic experience of Disconnection and Rejection is the core emotional need for Connection and Acceptance. This will enable the therapist to make a shift in the therapy to not just work to counteract a feeling of disconnection and rejection but also to work to develop and strengthen an experience of connection and acceptance informed by the elements that make up the need for connection and acceptance, which, in turn, will slowly strengthen the EASs associated with this need. With this clearer identification of categories of both core emotional needs and toxic experiences, parents and therapists are better positioned to know how to create the type of nurturing environment that promotes growth and how to avoid the toxic experiences that inhibit or derail it.

## 7. Limitations

For the four online samples, participants had to answer all the questions, and this may have affected their responses in some way. The online results were also obtained during the COVID-19 outbreak; this was a particularly stressful time, which may have also affected the results. The self-report methods used in this study also have their limitations. Since there were two methods of data collection, one online and the other in-person, this may have had an influence on the data as well. With the Singapore and Malaysia samples, incentives were offered, and this may have had an impact on the types of volunteers who chose to participate, limiting the generalizability of the findings. Lastly, although participants were drawn from countries where English was widely spoken, they were also more fluent in their own languages, so limitations may have been introduced by not having these questionnaires translated into their mother tongue. Unexpected results were encountered with the DT subscale of narcissism, and some items representing this subscale may need to be improved.

## 8. Future Studies

This study used six non-clinical community samples. Future studies should also focus on clinical samples. While this study had samples from the USA, Africa, and Asia, samples were lacking from Europe and South America, so it will be helpful for future studies to include samples from these parts of the world. Studies should also be performed to test the effects of the four categories of core emotional needs and toxic experiences with other measures of well-being and ill-being in order to assist in developing a fuller understanding of these patterns. This will be an important step in our efforts to assist parents and therapists in increasing their effectiveness in achieving better developmental and therapeutic outcomes.

## Figures and Tables

**Table 1:** The accompanying unmet need for each EMS, and the accompanying need for each EAS.

Second Order Domains of EMSs	Early Maladaptive Schemas (EMS)	Unmet Core Emotional Need/Toxic Experience	Second Order Domains of EASs	Early Adaptive Schemas (EAS)	Core Emotional Need/Psychological Nutrient
Disconnection and Rejection	Emotional Deprivation	Lack of nurturance, empathy, and protection	Connection and Acceptance	Emotional Fulfilment	Adequate nurturance, empathy, and protection
	Social Isolation/Alienation	Interference with or lack of support for connections with friends or groups outside of the home		Social Belonging	Facilitation/support of connections with friends and groups outside of the home
	Emotional Inhibition	Suppression of spontaneity		Emotional Openness and Spontaneity	Parents of significant others who are open, playful, expressive, and spontaneous
	Negativity/Pessimism	A persistent focus on the negative aspects of life		Healthy Self-Interest/Self-Care	Shown how to balance care for others and oneself
	Defectiveness/Shame	Criticism, rejection, and lack of acceptance			
	Mistrust/Abuse	Betrayal and lack of trust			
Impaired Autonomy and Performance	Dependence/Incompetence	Overprotection, lack of support for autonomy	Healthy Autonomy and Performance	Healthy Self-Reliance/Competence	Being believed in and supported for independent functioning and competence
	Enmeshment/Undeveloped Self	Intrusiveness, overinvolvement		Healthy Boundaries/Developed Self	Respect for autonomy, privacy, and making one’s own choices
	Abandonment/Instability	Lack of stability and/or reliability		Stable Attachment	Reliable and stable availability
	Vulnerability to Harm or Illness	Exaggerated fear of catastrophises			
	Failure	A sense of inadequacy in relation to one’s peers			
	Subjugation	Anger, retaliation, or abandonment for expression of needs of emotions			
Impaired Limits	Insufficient Self-Control/Self-Discipline	Inadequate limits	Reasonable Limits	Healthy Self-Control/Self-Discipline	Adequate limits
	Entitlement/Grandiosity	Spoiled, put on a pedestal, or rejected and shamed		Success	Adequate experiences of success
	Approval-Seeking/Recognition Seeking	Conditional acceptance or love			
Excessive Responsibility and Standards	Unrelenting Standards/Hypercriticalness	Pressured to meet unrealistic expectations	Healthy Standards and Reciprocity	Realistic Expectations	Balanced Sense of Work and Relaxation
	Punitiveness	Lack of forgiveness of self/others		Empathic Consideration	Sense of Reciprocity with Others
	Self-Sacrifice	Lack of consideration of one’s needs		Self-Compassion	Adequate Sense of Forgiving Self
				Self-Directedness	Adequate Sense of Self Appraisal
				Basic Health/Optimism	Adequate Sense of Optimism

**Table 2:** Demographic characteristics of samples from Singapore and Malaysia.

	Categories	Singapore	Malaysia
		*N* (%)	*N* (%)
Gender	Men	260 (41.20)	83 (35.78)
	Women	371 (58.80)	149 (64.22)
	Did not specify	0 (0.00)	0 (0.00)
Age (years)	20–29	100 (15.85)	42 (18.10)
	30–39	167 (26.47)	81 (34.91)
	40–49	277 (43.90)	90 (38.79)
	≥50	87 (13.79)	18 (7.79)
	Did not specify	0 (0.00)	1 (0.43)
Race	Chinese	508 (80.51)	205 (88.36)
	Indonesian	5 (0.79)	5 (2.16)
	Indian	15 (2.38)	3 (1.29)
	Filipino	91 (14.42)	9 (3.88)
	Caucasian/White	2 (0.32)	2 (0.86)
	Black	N. A.	N. A.
	Latino	N. A.	N. A.
	Asian	N. A.	N. A.
	Others	9 (1.43)	8 (3.45)
	Did not specify	1 (0.16)	0 (0.00)
Missing >10% values		3 (0.48)	3 (1.29)
Final Sample Size		628	229

**Table 3:** Demographic characteristics of online samples—the USA, South Africa, Nigeria, and India.

Characteristics	Categories	USA	South Africa	Nigeria	India
Gender	Men	147	159	209	169
	Women	249	231	155	137
	Did Not Specify	0	0	0	0
Age	Mean Age	43.69	42.11	45.7	42.39
	SD	9.12	6.79	7.19	7.67
Race	Chinese	N. A.	N. A.	N. A.	N. A.
	Indonesian	N. A.	N. A.	N. A.	N. A.
	Indian	N. A.	7	N. A.	N. A.
	Filipino	N. A.	N. A.	N. A.	N. A.
	Caucasian/White	104	65	N. A.	N. A.
	Black	52	135	N. A.	N. A.
	Latino	121	N. A.	N. A.	N. A.
	Asian	99	N. A.	N. A.	N. A.
	Mixed Race People	N. A.	17	N. A.	N. A.
	Yoruba	N. A.	N. A.	191	N. A.
	Ibo	N. A.	N. A.	72	N. A.
	Hausa	N. A.	N. A.	5	N. A.
	North India	N. A.	N. A.	N. A.	31
	East India	N. A.	N. A.	N. A.	44
	South India	N. A.	N. A.	N. A.	138
	West India	N. A.	N. A.	N. A.	45
	Others	20	7	96	48
	Did not specify	0	159	0	0
	Missing >10% values	0	0	0	0
	Final Sample Size	396	390	364	306

**Table 4:** CFA results for second order of EMSs.

Country	Model	No. of Parameters	χ^2^	*df*	*p*	χ^2^/*df*	CFI	TLI	RMSEA
USA	5-factor (Y)	568	6683.31	3887	<0.001	1.72	0.89	0.89	0.043 [0.041 0.044]
	4-factors (B)	564	6674.37	3891	<0.001	1.72	0.90	0.89	0.043 [0.041 0.044]
South Africa	5-factor (Y)	568	6550.75	3887	<0.001	1.69	0.93	0.93	0.042 [0.040 0.044]
	4-factors (B)	564	6576.98	3891	<0.001	1.69	0.93	0.93	0.042 [0.040 0.044]
Nigeria	5-factor (Y)	568	6340.86	3887	<0.001	1.63	0.93	0.93	0.042 [0.040 0.043]
	4-factors (B)	564	6302.13	3891	<0.001	1.62	0.93	0.93	0.041 [0.058 0.066]
India	5-factor (Y)	568	5868.55	3887	<0.001	1.51	0.91	0.91	0.041 [0.039 0.043]
	4-factors (B)	564	5852.39	3891	<0.001	1.50	0.91	0.91	0.041 [0.038 0.043]
Malaysia	5-factor (Y)	568	5593.59	3887	<0.001	1.44	0.90	0.90	0.044 [0.041 0.046]
	4-factors (B)	564	5593.10	3891	<0.001	1.44	0.90	0.90	0.044 [0.041 0.046]
Singapore	5-factor (Y)	568	9388.97	3887	<0.001	2.42	0.89	0.89	0.047 [0.046 0.049]
	4-factors (B)	564	9125.83	3891	<0.001	2.35	0.90	0.90	0.046 [0.044 0.048]

Five-factor (Y)—five-factor model from Young et al. [4]; four-factor (B)—four-factor model from Bach et al. [27].

**Table 5:** MGCFA results for four-factor model of Bach et al. [27] of EMSs using samples from the USA, South Africa, India, Nigeria, Singapore, and Malaysia.

Model	Number of Parameters	χ^2^	*df*	*p*	χ^2^/*df*	CFI	TLI	RMSEA [90% CI]		
Configural invariance	3314	39,537.01	23,416	<0.001	1.688	0.911	0.909	0.042 [0.042 0.043]	-	Accept
Metric invariance	2884	40,227.42	23,846	<0.001	1.687	0.909	0.909	0.042 [0.041 0.043]	Configural vs. Metric	Accept
		(1288.77)	(430)	<0.001		(0.002)	(<0.001)	(<0.001)		
Scalar invariance	1174	42,195.77	25,556	<0.001	1.651	0.908	0.914	0.041 [0.040 0.042]	Metric vs. Scalar	Accept
		(3791.16)	(1710)	(<0.001)		(0.001)	(−0.005)	(−0.001)		
Residual variance invariance	634	40,834.98	26,096	<0.001	1.565	0.919	0.925	0.038 [0.38 0.039]	Scalar vs. Residual	Accept
		(1425.18)	(540)	(<0.001)		(−0.011)	(−0.011)	(−0.003)		
Factor variance invariance	594	25,337.4	26,116	<0.001	0.970	0.925	0.931	0.037 [0.036 0.038]	Residual vs. Factor variance	Accept
		(58.37)	(20)	(<0.001)		(−0.006)	(−0.006)	(−0.001)		
Factor covariance invariance	584	36,325.49	26,146	<0.001	1.39	0.944	0.948	0.032 [0.031 0.033]	Factor variance vs. Factor covariance	Accept
		(97.49)	(30)	(<0.001)		(−0.019)	(−0.017)	(−0.005)		
Factor mean invariance	568	37,704.95	26,162	<0.001	1.44	0.936	0.941	0.034 [0.033 0.035]	Factor covariance vs. Factor mean	Accept
		(379.54)	(16)	(<0.001)		(0.008)	(0.007)	(0.002)		
Acceptance criteria for indices (differences)						>0.9	>0.9	<0.08		
						(<0.01)	(<0.01)	(<0.015)		

**Table 6:** CFA results for second order of EASs.

Country	Model	No of Parameters	χ^2^	*df*	*p*	*χ* ^2^ */df*	CFI	TLI	RMSEA
USA	4-factors	356	3164.285	1464	<0.001	2.16	0.89	0.89	0.054 [0.052 0.057]
South Africa	4-factors	356	3438.13	1464	<0.001	2.35	0.90	0.89	0.059 [0.056 0.061]
Nigeria	4-factors	356	3109.449	1464	<0.001	2.12	0.91	0.91	0.056 [0.053 0.058]
India	4-factors	356	2743.26	1464	<0.001	1.87	0.91	0.91	0.053 [0.050 0.057]
Malaysia	4-factors	353	2554.97	1464	<0.001	1.75	0.94	0.94	0.057 [0.053 0.061]
Singapore	4-factors	355	4315.388	1464	<0.001	2.95	0.95	0.95	0.056 [0.054 0.058]

**Table 7:** MGCFA results for four-factor model of EASs using samples from the USA, South Africa, India, Nigeria, Singapore, and Malaysia.

Model	Number of Parameters	χ^2^	df	*p*	χ^2^/df	CFI	TLI	RMSEA [90% CI]		
Configural invariance	2081	18,992.29	8839	<0.001	2.149	0.930	0.927	0.055 [0.054 0.056]	-	Accept
Metric invariance	1821	18,772.882	9099	<0.001	2.063	0.933	0.932	0.053 [0.051 0.054]	Configural vs. Metric	Accept
		(475.504)	(260)	(<0.001)		(−0.003)	(−0.005)	(−0.002)		
Scalar invariance	776	20,711.615	10,144	<0.001	2.042	0.927	0.933	0.052 [0.051 0.053]	Metric vs. Scalar	Accept
		(3240.348	(1045)	(<0.001)		(0.006)	(−0.001)	(−0.001)		
Residual variance invariance	426	21,249.277	10,494	<0.001	2.025	0.926	0.934	0.052 [0.051 0.053]	Scalar vs. Residual	Accept
		(1664.481)	(350)	(<0.001)		(0.001)	(−0.001)	(<0.001)		
Factor variance invariance	406	20,461.874	10,514	<0.001	1.946	0.931	0.939	0.050 [0.049 0.051]	Residual vs. Factor variance	Accept
		(102.890)	(20)	(0.001)		(−0.005)	(−0.005)	(−0.002)		
Factor covariance invariance	376	16,863.421	10,544	<0.001	1.60	0.956	0.962	0.039 [0.038 0.041]	Factor variance vs. Factor covariance	Accept
		(89.004)	(30)	(0.001)		(−0.025)	(−0.023)	(−0.011)		
Factor mean invariance	356	18,444.819	10,564	<0.001	1.75	0.945	0.952	0.044 [0.043 0.045]	Factor covariance vs. Factor mean	Reject
		(528.058)	(12)	(<0.001)		(0.011)	(0.010)	(0.005)		
Acceptance criteria for indices (differences)						>0.9	>0.9	<0.08		
						(<0.01)	(<0.01)	(<0.015)		

**Table 8:** Pearson’s correlation coefficients between four-factor model (second-order schema domains) of EMSs and EASs using combined sample of six countries (*n* = 2313; both scales measuring EASs and EMSs were administered to all six countries).

	Core Emotional Needs				Toxic Experiences			
	CA	HAP	RL	HSR	DR	IAP	IL	ERS
CA	1							
HAP	0.67 ***	1						
RL	0.67 ***	0.65 ***	1					
HRS	0.73 ***	0.67 ***	0.67 ***	1				
DR	−0.56 ***	−0.43 ***	−0.40 ***	−0.47 ***	1			
IAP	−0.49 ***	−0.56 ***	−0.55 ***	−0.51 ***	0.84 ***	1		
IL	−0.27 ***	−0.27 ***	−0.36 ***	−0.37 ***	0.69 ***	0.68 ***	1	
ERS	−0.24 ***	−0.16 ***	−0.11 ***	−0.34 ***	0.65 ***	0.61 ***	0.58 *	1

*** *p* < 0.001, * *p* < 0.05. Core emotional needs (second-order four-factor model of EASs); CA—Connection and Acceptance; HAP—Healthy Autonomy and Performance; Rl—Reasonable Limits; HSR—Healthy Standards and Reciprocity; toxic experiences (second-order four-factor model of EMSs); DR—Disconnection and Rejection; IAP—Impaired Autonomy and Performance; IL—Impaired Limits; ERS—Excessive Responsibility and Standards.

**Table 9:** Pearson’s correlation coefficients between core emotional needs (four-factor model of EASs); toxic experiences (four-factor model of EMS); and measures of personality, well-being, and ill-being using combined Singapore and Malaysia samples (*n* = 628, *n* = 229; samples from the other four countries were not administered with these measures).

	Core Emotional Needs				Toxic Experiences			
	CA	HAP	RL	HSR	DR	IAP	IL	ERS
IPIP Agreeableness	0.35 ***	0.22 ***	0.17 ***	0.22 ***	−0.33 ***	−0.18 ***	−0.20 ***	−0.01
IPIP Conscientiousness	0.28 ***	0.32 ***	0.46 ***	0.30 ***	−0.28 ***	−0.38 ***	−0.38 ***	−0.04
IPIP Extraversion	0.44 ***	0.20 ***	0.25 ***	0.19 ***	−0.36 ***	−0.24 ***	−0.03	−0.13 ***
IPIP Intellect	0.23 ***	0.13 ***	0.20 ***	0.12 ***	−0.16 ***	−0.20 ***	−0.06	−0.12 ***
IPIP Neuroticism	−0.40 ***	−0.43 ***	−0.35 ***	−0.48 ***	0.44 ***	0.45 ***	0.30 ***	0.29 ***
Gratitude	0.48 ***	0.42 ***	0.34 ***	0.38 ***	−0.44 ***	−0.38 ***	−0.27 ***	−0.12 ***
Depression	−0.45 ***	−0.41 ***	−0.42 ***	−0.43 ***	0.61 ***	0.58 ***	0.43 ***	0.34 ***
Anxiety	−0.32 ***	−0.41 ***	−0.26 ***	−0.33 ***	0.49 ***	0.54 ***	0.33 ***	0.34 ***
Stress	−0.41 ***	−0.39 ***	−0.36 ***	−0.47 ***	0.55 ***	0.54 ***	0.45 ***	0.39 ***
SWLS	0.50 ***	0.42 ***	0.39 ***	0.41 ***	−0.45 ***	−0.38 ***	−0.27 ***	−0.20 ***

*** *p* < 0.001.

**Table 10:** Pearson’s correlation coefficients between core emotional needs and toxic experiences with DT using combined samples of four countries (*n* = 1456; Singapore and Malaysia samples were not administered with the DT scale).

	Machiavellianism	Narcissism	Psychopathy
Machiavellianism	1		
Narcissism	0.33 ***	1	
Psychopathy	0.48 ***	0.35 ***	1
Core Emotional Needs			
CA	−0.10 ***	0.24 ***	−0.16 ***
HAP	−0.14 ***	0.03	−0.24 ***
RL	−0.09 ***	0.18 ***	−0.20 ***
HSR	−0.08 **	0.04	−0.21 ***
Toxic Experiences			
DR	0.46 ***	−0.01	0.41 ***
IAP	0.41 ***	0.02	0.41 ***
IL	0.53 ***	0.27 ***	0.45 ***
ERS	0.34 ***	0.12 ***	0.25 ***

*** *p* < 0.001, ** *p* < 0.01.

**Table 11:** Linear regression results for effects of categories of core emotional needs and measures of IPIP personality traits, well-being, and ill-being using Singapore and Malaysia samples (*n* = 628, *n* = 229; samples from the other four countries were not administered with these measures).

Dependent Variable	Intercept.	CA	HAP	RL	HSR	R_sq	F_stat
IPIP Agreeableness	11.25 ***	1.33 ***	−0.03	−0.19	−0.13	0.13	30.52 ***
IPIP Conscientiousness	8.83 ***	−0.19	0.25	1.49 ***	−0.09	0.22	58.10 ***
IPIP Extraversion	5.71 ***	2.74 ***	−0.57 **	0.26	−1.02 ***	0.24	67.54 ***
IPIP Intellect	10.58 ***	0.98 ***	−0.26	0.51 **	−0.46 *	0.07	15.34 ***
IPIP Neuroticism	20.30 ***	−0.21	−0.55 **	0.05	−1.31 ***	0.25	68.89 ***
Gratitude	21.45 ***	2.28 ***	0.91 ***	−0.03	−0.01	0.25	69.44 ***
DAS Depression	16.85 ***	−1.14 ***	−0.31	−0.74 ***	−0.63 *	0.24	67.81 ***
DAS Anxiety	13.63 ***	−0.27	−1.62 ***	0.21	−0.28	0.17	44.96 ***
DAS Stress	18.19 ***	−0.48	−0.37	−0.14	−1.71 ***	0.23	64.86 ***
SWLS	5.36 ***	2.89 ***	0.66	0.60 *	0.08	0.26	76.51 ***

*** *p* < 0.001, ** *p* < 0.01, * *p* < 0.05.

**Table 12:** Linear regression results for effects of core emotional needs on and measures of IPIP personality traits, well-being, and ill-being using Singapore and Malaysia samples (*n* = 628, *n* = 229; samples from the other four countries were not administered with these measures).

Dependent Variable	Intercept.	Core Emotional Needs	R_sq	F_stat
IPIP Agreeableness	11.43 ***	0.94 ***	0.07	67.69 ***
IPIP Conscientiousness	8.15 ***	1.55 ***	0.15	156.04 ***
IPIP Extraversion	5.49 ***	1.41 ***	0.10	91.74 ***
IPIP Intellect	10.28 ***	0.80 ***	0.04	33.88 ***
IPIP Neuroticism	20.00 ***	−1.97 ***	0.22	245.42 ***
Gratitude	21.76 ***	3.15 ***	0.21	223.71 ***
DASDepression	17.07 ***	−2.83 ***	0.24	270.61 ***
DASAnxiety	12.96 ***	−1.95 ***	0.14	141.91 ***
DASStress	17.94 ***	−2.63 ***	0.22	235.32 ***
SWLS	5.47 ***	4.22 ***	0.24	273.98 ***

*** *p* < 0.001.

**Table 13:** Linear regression results for categories of toxic experiences and measures of IPIP personality traits, well-being, and ill-being using Singapore and Malaysia samples (*n* = 628, *n* = 229); samples from the other four countries were not administered with these measures).

Dependent Variable	Intercept.	DR	IAP	IL	ERS	R_sq	F_stat
IPIP Agreeableness	16.35 ***	−0.37 ***	0.12 **	−0.06 *	0.20 ***	0.17	44.32 ***
IPIP Conscientiousness	17.66 ***	0.03	−0.30 ***	−0.25 ***	0.24 ***	0.24	66.01 ***
IPIP Extraversion	12.78 ***	−0.50 ***	0.01	0.26 ***	0.07	0.2	51.74 ***
IPIP Intellect	15.19 ***	−0.03	−0.19 ***	0.10 **	−0.02	0.05	10.90 ***
IPIP Neuroticism	6.64 ***	0.17 ***	0.23 ***	−0.01	0.01	0.22	61.35 ***
Gratitude	39.53 ***	−0.59 ***	−0.18 **	−0.02	0.34 ***	0.24	65.41 ***
DAS Depression	−3.62 ***	0.47 ***	0.29 ***	0.04	−0.10 *	0.4	142.77 ***
DAS Anxiety	−2.58 ***	0.17 ***	0.43 ***	−0.05	0.03	0.31	94.10 ***
DAS Stress	−2.80 ***	0.29 ***	0.24 ***	0.14 **	0.05	0.34	109.68 ***
SWLS	30.79 ***	−0.68 ***	−0.20 *	0.04	0.19 **	0.21	56.12 ***

*** *p* < 0.001, ** *p* < 0.01, * *p* < 0.05.

**Table 14:** Linear regression results for toxic experiences and measures of IPIP personality traits, well-being, and ill-being using Singapore and Malaysia samples (*n* = 628, *n* = 229); samples from the other four countries were not administered with these measures).

Dependent Variable	Intercept	Toxic Experiences	R_sq	F_stat
IPIP Agreeableness	17.91 ***	−0.18 ***	0.05	43.56 ***
IPIP Conscientiousness	18.85 ***	−0.30 ***	0.10	97.79 ***
IPIP Extraversion	14.82 ***	−0.24 ***	0.05	47.85 ***
IPIP Intellect	15.73 ***	−0.15 ***	0.02	21.74 ***
IPIP Neuroticism	5.62 ***	0.43 ***	0.20	210.37 ***
Gratitude	43.11 ***	−0.58 ***	0.13	126.15 ***
DASDepression	−6.02 ***	0.79 ***	0.34	442.92 ***
DASAnxiety	−3.99 ***	0.61 ***	0.26	300.47 ***
DASStress	−3.88 ***	0.76 ***	0.33	421.42 ***
SWLS	34.07 ***	−0.77 ***	0.15	151.68 ***

*** *p* < 0.001.

**Table 15:** Linear regression results of categories of core emotional needs on Dark Triad (*n* = 1456; Singapore and Malaysia samples were not administered with the DT scale).

Dependent Variable	Predictors (Second-Order EASs)				R^2^	F_stat	N
	CA	HAP	RL	HSR			
Machiavellianism	0.00	−0.03 ***	0.00	0.00	0.02	7.06 ***	1456
Narcissism	0.08 ***	−0.06 ***	0.08 ***	−0.04 ***	0.12	49.89 ***	1456
Psychopathy	0.01	−0.04 ***	−0.02	−0.01 *	0.07	25.90 ***	1456

*** *p* < 0.001, * *p* < 0.05.

**Table 16:** Linear regression results of core emotional needs on Dark Triad (*n* = 1456; Singapore and Malaysia samples were not administered with the DT scale).

Dependent Variable	Intercept.	Core Emotional Needs	R^2^	F_stat
Machiavellianism	3.11 ***	−0.03 ***	0.01	19.87 ***
Narcissism	2.21 ***	0.03 ***	0.02	26.27 ***
Psychopathy	2.61 ***	−0.05 ***	0.05	83.47 ***

*** *p* < 0.001.

**Table 17:** Linear regression results of categories of toxic experiences on Dark Triad (*n* = 1456; Singapore and Malaysia samples were not administered with the DT scale).

Dependent Variable	Predictors (Second-Order EMSs)				R^2^	F_stat	N
	DR	IAP	IL	ERS			
Machiavellianism	0.30 ***	−0.20 **	0.29 ***	−0.02	0.26	33.82 ***	1456
Narcissism	−0.32 ***	−0.19 **	0.42 ***	0.13 **	0.22	26.92 ***	1456
Psychopathy	0.14 **	−0.12 *	0.21 ***	−0.05	0.15	17.68 ***	1456

*** *p* < 0.001, ** *p* < 0.01, * *p* < 0.05.

**Table 18:** Linear regression results of toxic experiences on Dark Triad (*n* = 1456; Singapore and Malaysia samples were not administered with the DT scale).

Dependent Variable	Intercept.	Toxic Experiences	R^2^	F_stat
Machiavellianism	1.52 ***	0.42 ***	0.24	465.53 ***
Narcissism	2.49 ***	0.09 ***	0.01	19.45 ***
Psychopathy	0.98 ***	0.31 ***	0.19	336.83 ***

*** *p* < 0.001.

## Data Availability

The data underlying the results presented in the study are available from Rakesh Rai, who can be reached at rakesh.kr@ctsnet.co.
